# Determinants of TRPV4 Activity following Selective Activation by Small Molecule Agonist GSK1016790A

**DOI:** 10.1371/journal.pone.0016713

**Published:** 2011-02-14

**Authors:** Min Jin, Zizhen Wu, Ling Chen, Jose Jaimes, Diana Collins, Edgar T. Walters, Roger G. O'Neil

**Affiliations:** Department of Integrative Biology and Pharmacology, The University of Texas Health Science Center, Houston, Texas, United States of America; University of São Paulo, Brazil

## Abstract

TRPV4 (Transient Receptor Potential Vanilloid 4) channels are activated by a wide range of stimuli, including hypotonic stress, non-noxious heat and mechanical stress and some small molecule agonists (e.g. phorbol ester 4α-PDD). GSK1016790A (GSK101) is a recently discovered specific small molecule agonist of TRPV4. Its effects on physical determinants of TRPV4 activity were evaluated in HeLa cells transiently transfected with TRPV4 (HeLa-TRPV4). GSK101 (10 nM) causes a TRPV4 specific Ca^2+^ influx in HeLa-TRPV4 cells, but not in control transfected cells, which can be inhibited by ruthenium red and Ca^2+^-free medium more significantly at the early stage of the activation rather than the late stage, reflecting apparent partial desensitization. Western blot analysis showed that GSK101 activation did not induce an increase in TRPV4 expression at the plasma membrane, but caused an immediate and sustained downregulation of TRPV4 on the plasma membrane in HeLa-TRPV4 cells. Patch clamp analysis also revealed an early partial desensitization of the channel which was Ca^2+^-independent. FRET analysis of TRPV4 subunit assembly demonstrated that the GSK101-induced TRPV4 channel activation/desensitization was not due to alterations in homotetrameric channel formation on the plasma membrane. It is concluded that GSK101 specifically activates TRPV4 channels, leading to a rapid partial desensitization and downregulation of the channel expression on the plasma membrane. TRPV4 subunit assembly appears to occur during trafficking from the ER/Golgi to the plasma membrane and is not altered by agonist stimulation.

## Introduction

TRPV4 is a non-selective Ca^2+^ - permeable cation channel that belongs to the TRP superfamily. It is ubiquitously expressed in various tissues such as renal epithelia, lung epithelia, vascular endothelia, and nervous systems [1]–[4]. Studies show that TRPV4 is activated by hypotonic stress, moderate heat, mechanical stress, phorbol ester (4α-PDD) and arachidonic acid metabolites [5]–[14].

GSK101 (GSK1016790A) is a novel activator of TRPV4, which has been shown to be a more specific and potent activator (at nanomolarlevels) as compared to the traditional 4α-PDD[15], [16]. Recent studies show that GSK101 stimulates TRPV4 in multiple cell types including endothelial cells, urinary smooth muscle cells, urothelial cells and HEK-293 cells over-expressing TRPV4 [15]–[18]. Being a novel TRPV4 agonist, the signaling pathway of GSK101 is not well understood. In addition to the various signaling pathways that may modulate the channel activity, ion channel activation also involves subunit assembly/disassembly, trafficking, insertion and endocytosis of functional channel to/from the plasma membrane. Limited studies on TRP channel trafficking have shown, however, that some stimuli can cause the exocytosis and insertion of the channel into the plasma membrane, thus contributing to channel activity[19]–[23], while other studies have shown that TRPV4 is down regulated under angiotensin stimulation in rat smooth muscle cells [24]. It has also been demonstrated that TRPV4 channels at the plasma membrane typically reflect a homotetrameric assembly [25], [26], but heterotetramer structures can form with other TRP family isoforms which lead to altered channel function [27], [28]. Indeed, a recent study of TRPP2 subunit structure demonstrated that subunit disassembly might be an important component of channel inactivation [29], [30]. Hence, the activity of TRPV4 at the plasma membrane is likely a dynamic process reflecting both abundance and subunit assembly. This regulation may, of course, also include the role of more traditional modulating pathways, including both phosphorylation and nitrosylation events, which can contribute to channel regulation [31], [32].

In this study, we set out to investigate the relationship between GSK101-induced TRPV4 activation and its expression and subunit assembly at the plasma membrane as the physical determinants of TRPV4 activity. It was found that agonist stimulation did not alter the apparent subunit assembly within the plasma membrane, but it induced an early rapid downregulation of TRPV4 expression at the plasma membrane that was associated with a rapid desensitization of the TRPV4 channel in a Ca^2+^-independent manner.

## Results

### GSK101 stimulates Ca^2+^ influx in HeLa-TRPV4 cells

RT-PCR using HeLa cell mRNA and primer pairs designed for TRPV channels showed that HeLa cells do not expressed TRPV4 channels at the mRNA level. Likewise, Western blot using anti-TRPV4 antibody also could not detect TRPV4 at the protein level in wild type HeLa cells (data not shown). Therefore, HeLa cells were used as an overexpression cell model when transiently transfected with a TRPV4 containing plasmid similar to that done previously in HEK and CHO cells [5], [13].

**Figure 1:**
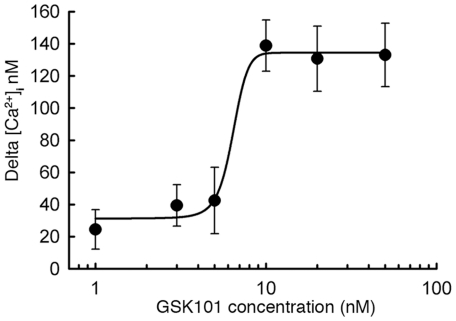
Dose-dependent activation of Ca^2+^ influx under GSK101 stimulation. HeLa-TRPV4 cells were stimulated with GSK101 at 1, 3, 5, 10, 20 and 100 nM. Delta [Ca^2+^]_i_ was determined by calcium imaging and the dose-response curve was fitted by a sigmoidal dose-response function using SigmaPlot 10.0. The calculated EC_50_ = 3.3 nM.

In HeLa-TRPV4 cells, Ca^2+^ influx was stimulated by GSK101 in a dose-dependent manner as shown in Figure 1. The GSK101 - Ca^2+^ influx relationship could be fitted by asigmoidal dose-responsefunction, which yielded an EC_50_ of 3.3 nM for GSK101 stimulation. GSK101 at 10 nM displayed a near maximum stimulation and was, therefore, used as the preferred concentration to activate TRPV4 channels in this study.

**Figure 2:**
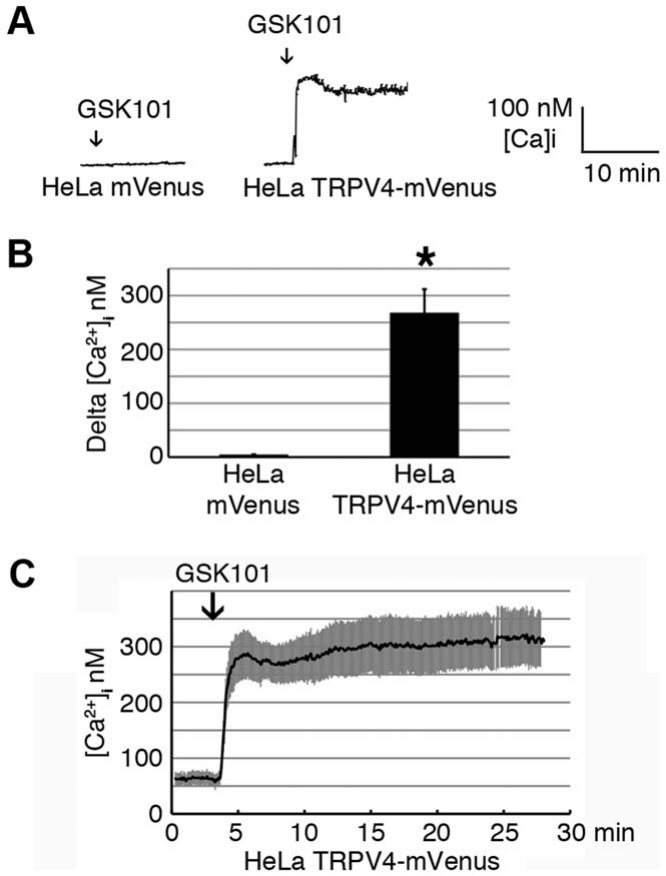
Effects of 10 nM GSK101 on intracellular Ca^2+^ concentration in HeLa-TRPV4 cells using calcium imaging analysis. A. The representative Ca^2+^ trace of HeLa cells (average of 15-20 cells from one experiment) transiently transfected with the empty mVenus-N1 vector (HeLamVenus) showed no response to GSK101 stimulation (negative control). The representative Ca^2+^ trace of HeLa TRPV4-mVenus cells showed a rapid increase in intracellular Ca^2+^ concentration under GSK101 stimulation, reaching its maximum level within 2 minutes and sustained for over 30 min. B.Summary bar graph showing statistically significant difference in delta [Ca^2+^]_i_ between HeLamVenus cells and HeLa TRPV4-mVenus cells (Mean ± SE, n = 3–6, P<0.05) under GSK101 stimulation. ***C*** **.** The averaged time course showing the effect of GSK101 on intracellularCa^2+^ concentration in HeLa TRPV4-mVenus (Mean ± SE, n = 8).

In order to test the specificity of GSK101 on TRPV4 activation, HeLa cells transfected with mVenus-N1 vector were compared with HeLa cells transfected with TRPV4-mVenus. Only cells positively identified as expressing TRPV4-mVenus (via excitation at 488-nm wavelength) were chosen for the study. GSK101 did not cause an increase in intracellular Ca^2+^ concentration in HeLa cells transfected with mVenus-N1 vector, while HeLa cells transfected with TRPV4-mVenus responded rapidly to GSK101 with an immediate increase in intracellular Ca^2+^ concentration (Figure 2A). As shown in Figure 2B, the change in intracellular Ca^2+^ in HeLa TRPV4-mVenus cells averaged 267.5±44.3 nM (n = 6) while a significant change was not detected in HeLamVenus cells (4.2±1.1 nM, n = 3). This result further confirmed that GSK101 is a specific activator of TRPV4 that induces Ca^2+^ influx intoHeLa TRPV4-mVenus (HeLa-TRPV4) cells.

The effect of GSK101 on intracellular Ca^2+^ in HeLa-TRPV4 cells was sustained over many minutes. While GSK101 induced an early peak Ca^2+^ influx, the intracellular Ca^2+^ levels only displayed a modest decay over time in the continued presence of the agonist, as shown by the average time course of the response in Figure 2C (n = 8). The increase in intracellular Ca^2+^ concentration in HeLa-TRPV4 reached its peak at 1.9±0.3 minutes (n = 8) and was maintained for over 30 minutes.

**Figure 3:**
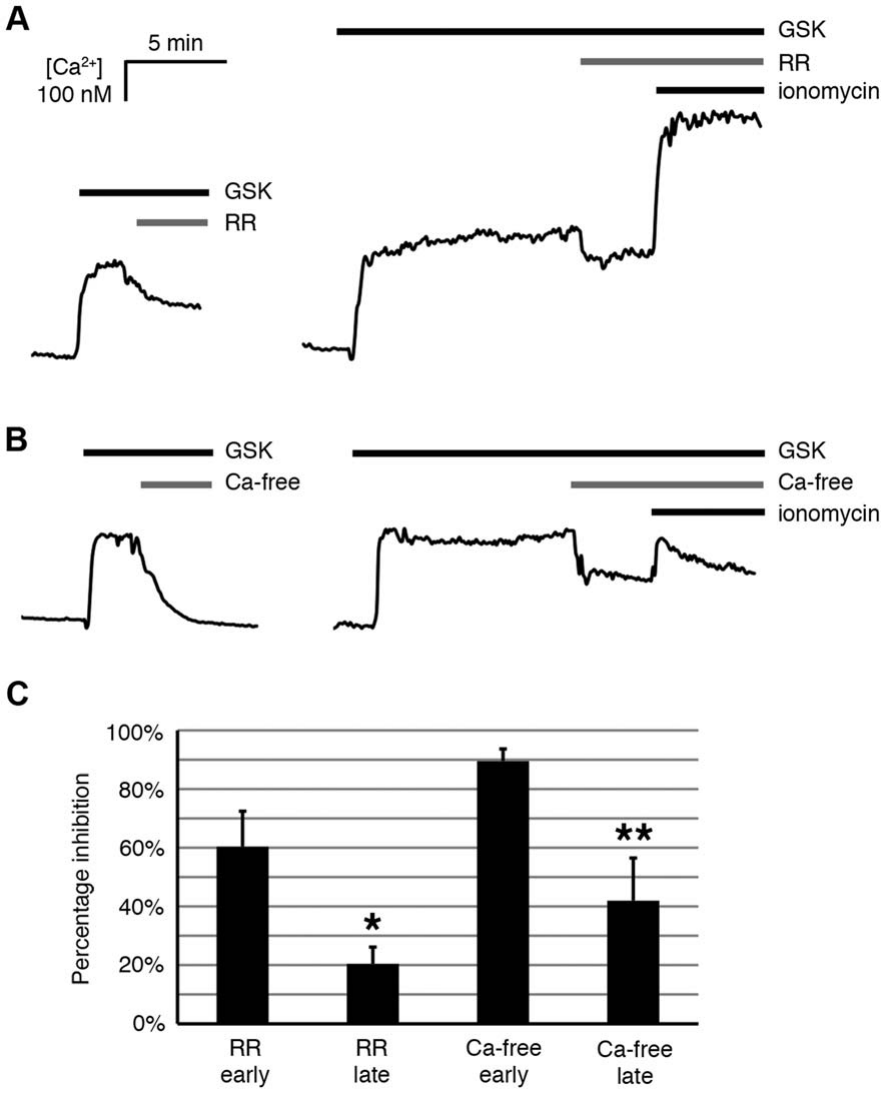
Effects of ruthenium red (3 µM) and Ca^2+^ free media on GSK101-induced intracellular Ca^2+^ elevation in HeLa-TRPV4 cells. A. Ruthenium red (RR) at 3 µM partially inhibited GSK101-induced intracellular Ca^2+^ elevation when added early (1–3 min post stimulation), while having less inhibitory effect if added at a later time point (10 min post stimulation). Ionomycin (5 µM) added on top of RR caused a significant Ca^2+^ influx in these cells. B. Ca^2+^ free media abolished GSK101-induced intracellular Ca^2+^ elevation when added early (3 min post stimulation), while having a much smaller effect if added at a later time point (10 min post stimulation). Ionomycin (5 µM) added while the cells were in Ca^2+^ free media caused a transient increase in intracellular Ca^2+^ concentration followed by the trending down of intracellular Ca^2+^. C.Summary bar graph showing the percentage inhibition of RR and Ca^2+^ free media added at early or late stage of GSK101 activation, respectively. The inhibition at the late stage displayed a much smaller percentage inhibition for either RR or Ca^2+^ free media(Mean ± SE, P<0.05, n = 4–7). * Significant difference between early and late inhibition by RR; ** significant difference between early and late inhibition by Ca^2+^ free media.

To identify whether the increase in intracellular Ca^2+^ concentration came from extracellular sources and/or intracellular stores, ruthenium red (RR), a TRPV channel inhibitor, and Ca^2+^-free media were utilized in calcium imaging experiments. As shown in Figure 3A, RR (3 µM) partially inhibited GSK101-induced Ca^2+^ influx. RR was more potent when added at an early stage of GSK101 activation (1–3 min post GSK101 activation). The inhibition by RR was reduced if added at a later time point (10 min post GSK101 activation). Addition of ionomycin, a Ca^2+^ionophore, on top of RR still caused a significant increase in intracellular Ca^2+^, indicating that fura 2 was not sequestered and fura 2 signaling was still active (Figure 3A). Figure 3B demonstrates that the GSK101-induced increase in intracellular Ca^2+^ was almost abolished immediately after replacing the extracellular bathing solution with a Ca^2+^-free solution, but only if the Ca^2+^-free media was added near the peak of the GSK101-induced responses (3 min post GSK101 activation). Similarly, GSK101/Ca^2+^-free medium only modestly diminished GSK101-induced intracellular Ca^2+^ levels when the GSK101/Ca^2+^-free medium was applied at 10 min post GSK101activation. Ionomycin added on top of the Ca^2+^-free medium caused a moderate but transient increase in intracellular Ca^2+^ that was quickly diminished due to the depletion of Ca^2+^ in the environment (Figure 3B). Figure 3C shows that there was a significant difference between the early and late stages of inhibition by either RR or Ca^2+^-free media, as demonstrated by the normalized percentage inhibition (n = 4–7, Figure 3C). This likely indicates that the increase in intracellular Ca^2+^ concentration was mainly due to Ca^2+^ influx through the TRPV4 channels at the early stage of the activation, and that the TRPV4 channel appeared to have partially desensitized following an extended activation period. The reason for the continued elevated Ca^2+^ levels under these conditions is not known, but likely reflects changes in other pathways of Ca^2+^ entry/exit, such as release from stores, or inhibition of Ca^2+^ efflux from the cytoplasm.

### GSK101 activates TRPV4 channel in whole-cell patch clamp analysis

**Figure 4:**
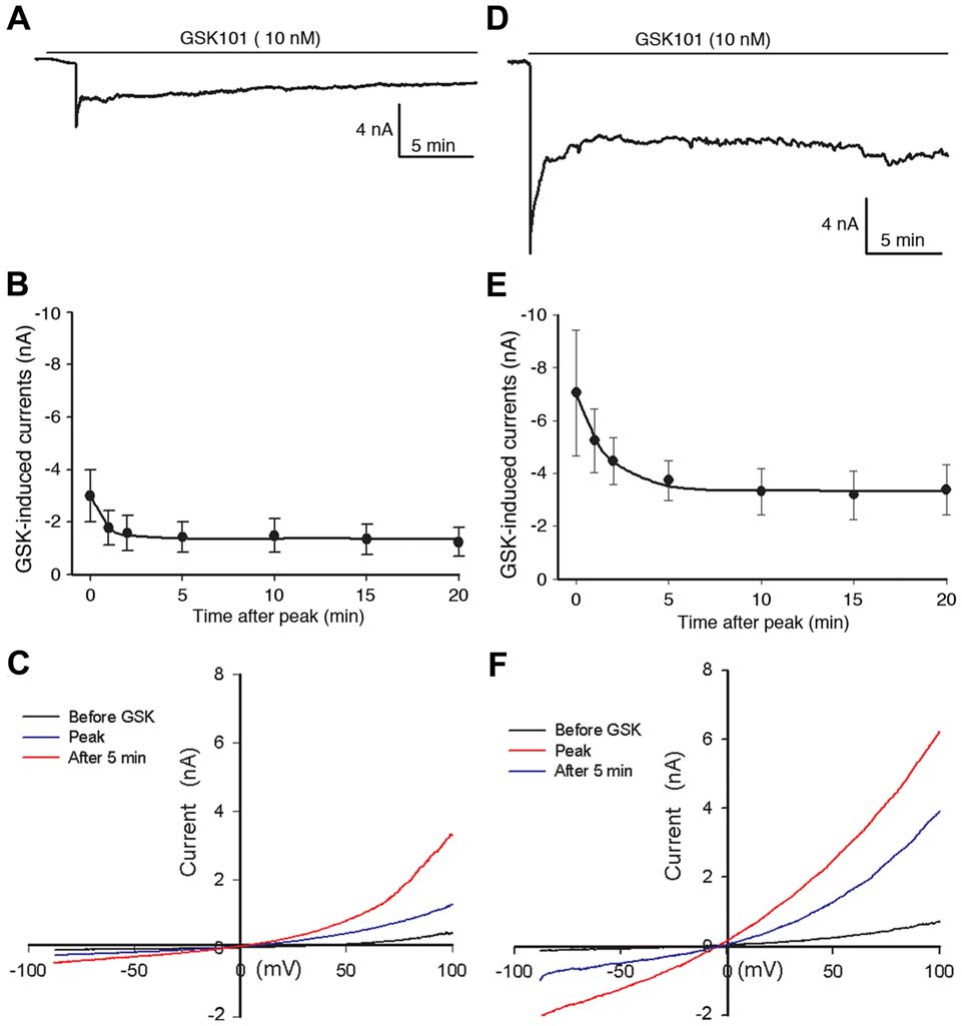
Effects of GSK101 on whole-cell membrane current (patch clamp) in HeLa-TRPV4 cells. A–C: HeLa-TRPV4 cells in Ca^2+^ containing media. A. A representative trace of GSK101-induced whole cell current showing a rapid increase at 1 min stimulation followed by channel desensitization (holding potential at −90 mV). B**.** Summary data showing the time course of GSK101-induced membrane current (Mean ± SE, n = 4) displaying a peak activation at 1 min followed by a partial desensitization of the channels. The time course for desensitization was fit to a single exponential equation and yielded a time constant (τ) for decay of 0.8±0.1 minutes.C**.** Current-voltage plot of a HeLa-TRPV4 cell under GSK101 stimulation, using a voltage ramp protocol from −100 to 100 mV over 200 msec. ***D–E***: HeLa-TRPV4 cells in Ca^2+^ free media (Na^+^ containing media). D. A representative trace of GSK101-induced whole cell current showing a similar pattern of channel activation as shown in A, but at a larger magnitude in Ca^2+^ free media. E. Summary data showing the time course of GSK101-induced membrane current (Mean ± SE, n = 4) in Ca^2+^ free media. The time course for desensitization was fit to a single exponential equation witha τ for decay of 3.8±1.8 minutes (NS from Ca^2+^-containing media in B). F. Current-voltage plot of a HeLa-TRPV4 cell under GSK101 stimulation in Ca^2+^ free media, using a voltage ramp protocol from −100 to 100 mV over 200 msec.

Although calcium imaging detected significant Ca^2+^ influx in response to GSK101 in HeLa-TRPV4 cells, it was necessary to determine the specific role of TRPV4 channels in regulating intracellular Ca^2+^ levels. The whole cell patch clamp technique was utilized to study the channel currents under GSK101 stimulation using either Ca^2+^-containing or Ca^2+^-free media in HeLa-TRPV4 cells. Whole-cell recordings in Ca^2+^-containing media are shown in Figure 4A–C. A representative current trace following GSK101 stimulation is shown in Figure 4A. At a holding potential of −90 mV, the GSK101-induced current occurredimmediately, reached a peak current at 1 min after activation, and relaxed to a pseudosteady state for the remainder of the recording (20 min). A similar time course was observed for four separate cells, with each demonstrating an early rapid activation, a peak near 1 min, followed by an apparent partial desensitization of the channel (Figure 4B). To further characterize the GSK101-induced current, a whole-cell current-voltage relation was generated using a voltage ramp protocol (−100 mV to +100 mV) over 200 msec duration. The IV-plot shows the GSK101-induced inward and outward currents, including the peak current at 1 min and the expected decay at the 5-min time pointin HeLa-TRPV4 cells. The characteristic outward rectification for TRPV4-currents at positive voltages is readily apparent (Figure 4C). Figure 4D–F shows the whole cell recording obtained in Ca^2+^-free media where Na^+^ is the dominant charge carry for this non-selective cation channel. The GSK101-induced currents in Ca^2+^-free media displayed similar patterns. This demonstrates that in the absence of Ca^2+^ in the media, Na^+^ readily permeated the TRPV4 channels under GSK101 stimulation causing even larger transmembrane currents. Similar current-voltage relationship under GSK101 stimulation was observed by others in TRPV4-expressing HEK-293 cells and human aortic endothelial cells [15], [16]. These electrophysiological findings indicate that the Ca^2+^ influx detected by calcium imaging during the first 3 minutes in HeLa-TRPV4 cells was likely primarily through TRPV4 channels. Moreover, the desensitization of TRPV4 under prolonged GSK101 stimulation did not appear to be calcium-dependent since it still occurred in calcium-free media.

### GSK101 down-regulates TRPV4 expression on plasma membrane in HeLa-TRPV4 cells

**Figure 5:**
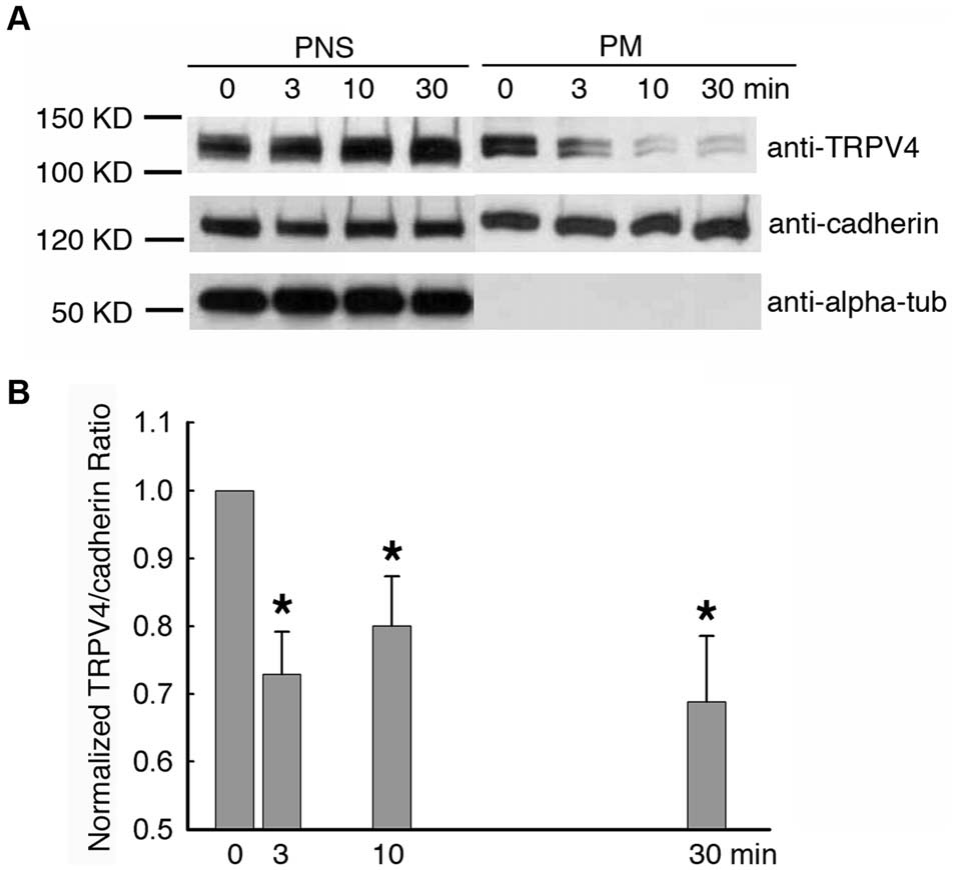
Effect of GSK101 on the plasma membrane expression of TRPV4 channel in HeLa-TRPV4 cells, over the time course of 30 min. A. Western blots showing that GSK101 caused a downregulation of TRPV4 in the pure plasma membrane fractions, while having no significant effect on the TRPV4 abundance in the post-nuclear (whole cell) lysates. B. Densitometry of the normalized TRPV4/cadherin ratio on the plasma membrane showed the statistically significant downregulation of TRPV4 under GSK101 treatment in HeLa-TRPV4 cells (Mean ± SE, n = 5, P<0.05).

To define the factors that contribute to GSK101-induced TRPV4 channel activation, the expression of TRPV4 on the plasma membrane was investigated using Western blot analysis. For many years a biotin-streptavidin system has been commonly used to isolate the plasma membrane fraction from whole cells and tissues. However, our study showed that the plasma membrane fraction acquired from the biotin-streptavidin system inevitably contained cytosolic proteins, for example cytoskeleton proteins (e.g. actin and tubulin), indicating this method is not ideal for studying pure plasma membrane proteins (data not shown). Instead, a sucrose-gradient based membrane protein extraction kit (Biovision, Inc., Mountain View, CA) showed very clean preparations of plasma membrane proteins without cytosolic contaminants, which was confirmed by non-detectable tubulin levels using anti-alpha tubulin antibody in Western blots (Figure 5).

HeLa-TRPV4 cells were harvested and plasma membrane proteins purified at 0, 3, 10 and 30 min time points under GSK101 stimulation. The lysates were then probed with anti-TRPV4 antibody to test the channel expression. Wild type TRPV4 has a predicted molecular weight of 98 kDa; the TRPV4-mVenus then has a predicted molecular weight of 123 KD due to the 25 kDa tag. The lysates were also probed with anti-pan cadherin for loading control and with anti-alpha tubulin for detecting cytosolic contamination (Figure 5A). Densitometry was preformed by scanning the TRPV4 and pan-cadherin protein levels, and the normalized ratios of TRPV4/pan-cadherin were calculated for GSK101 treatment at 0, 3, 10 and 30 min (Figure 5B). It is shown that 10 nM GSK101 caused an early and statistically significant (P<0.05, n = 5) down-regulation of TRPV4 on HeLa-TRPV4 cell membrane within 3 min of GSK101 stimulation. It is apparent that the plasma membrane TRPV4 abundance decreased by nearly 30% at 3 minutes post stimulation and then remained depressed for the remaining period of GSK101 stimulation. This early downregulation appeared to parallel the early desensitization of the channel demonstrated in the patch clamp studies (Figure 4). In summary, GSK101 stimulation appears to lead to an early rapid activation of TRPV4 at the plasma membrane, generating high levels of Ca^2+^ influx, followed by channel endocytosis from the plasma membrane that may contribute to the channel desensitization.

### FRET analysis HeLa-TRPV4 cells under GSK activation

Recently Shigematsu and coworkers have discovered that His-tagged rat TRPV4 forms a tetramer by using chemical cross-linking and Nanogold labeling in transmission electron microscopy [33]. They have also successfully reconstructed the 3D structure of the TRPV4 tetramer using single-particle cryoelectron microscopy. In the current study, we have demonstrated that GSK101 activates the TRPV4 channel within 1 to 3 min of stimulation. It was anticipated that the immediate activation of TRPV4 by GSK101 could include either an increase in the number of TRPV4 channels or the assembly of functional tetramers at the plasma membrane or both. Our western blots using purified plasma membranes suggested that it is unlikely that the number of TRPV4 channels on the plasma membrane increased under GSK101 activation. Therefore FRET was utilized in an attempt to look for the changes in the association among TRPV4 subunits while forming functional homotetrameric channels where an increase in FRET would be consistent with an increase in homotetrameric assembly and a decrease in homotetrameric disassembly.

**Figure 6:**
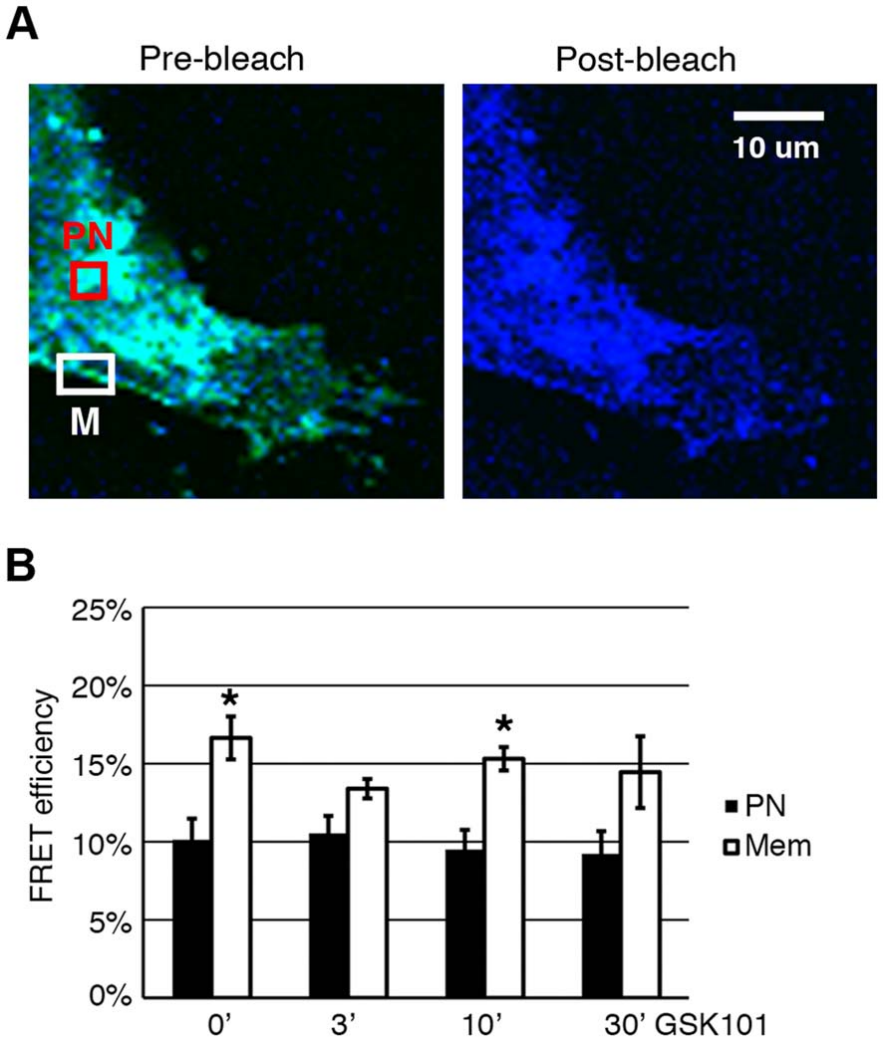
FRET analysis of TRPV4 channel under GSK101 (10 nM) treatment. HeLa cells were co-transfected with TRPV4-mCerulean (donor, in blue) and TRPV4-mVenus (acceptor, in yellow), and fixed at 0, 3, 10 and 30 min after GSK101 treatment. A**.** Pre-bleach and post-bleach images of TRPV4-mCerulean and TRPV4-mVenus overlapping with each other. Post-bleach image lost TRPV4-mVenus (from laser bleaching at 514 nm) and appeared bluer due to the increase of energy in TRPV4-mCerulean. The red box PN represented the perinuclearROI, the white box M represented the plasma membrane ROI. B. Summary of FRET efficiency between TRPV4-mCerulean and TRPV4-mVenus under three independent experiment sets of GSK101 stimulation. There was no significant different in FRET efficiency among the time point groups, both in PN and M regions (Mean ± SE, n = 3, P>0.05). However, FRET efficiency was significantly higher in the M region than in the PN region, in 0 and 10 min post GSK treatment groups (Mean ± SE, n = 3, P<0.05).

HeLa cells were transiently co-transfected with TRPV4-mCerulean (donor) and TRPV4-mVenus (acceptor). The cells were fixed at four time points of GSK101 treatments, 0, 3, 10 and 30 min. A widely used FRET photo-bleaching protocol was used to bleach the TRPV4-mVenus with the 514 nm laser, the changes in fluorescence intensity in TRPV4-mCerulean were recorded and analyzed. Figure 6A shows the pre-bleach and post-bleach images of TRPV4-mCerulean and TRPV4-mVenus, with the illustrated Region of Interest (ROI) for perinuclear (PN) and membranous (M) regions. Statistical analysis using 50 to 80 ROI for perinuclear and membranous regions (from 3 independent experiment sets) were performed for GSK101 treatment and FRET efficiency was calculated. For the plasma membrane ROI, the FRET efficiencies did not differ significantly among the membranous ROI from different treatment groups, indicating that GSK101 does not cause channel activation by alterations in the relative location/association of homotetrameric complexes. It was also noted in Figure 6B that the FRET efficiency of TRPV4 constructs at the plasma membrane tended to be higher (near 15%) than those in the PN regions (near 10%). Indeed, at 0 and 10 min of GSK101 treatment the FRET efficiency of TRPV4 on the plasma membrane was significantly higher than the perinuclear population of TRPV4 (n = 3, P<0.05). This suggested that TRPV4 monomers were not fully assembled into homotetramers in the PN region and that the mature tetrameric structure of the TRPV4 channel likely forms during the trafficking between the ER/Golgi and the plasma membrane [25].

## Discussion

This study evaluated the determinants of TRPV4 activity in a TRPV4 over-expression system, HeLa-TRPV4 cells. Our calcium imaging studies show that the small molecule agonist, GSK101, causes an early and rapid activation of TRPV4, leading to Ca^2+^ influx in HeLa-TRPV4 cells, but not in control transfected HeLa-mVenus cells. To be more specific, it was observed that the early rise in intracellular Ca^2+^ levels reached a peak in 1.9 minutes and was sustained thereafter in HeLa-TRPV4 cells.

It is of note that HeLa-TRPV4 cells did not return the GSK101-induced intracellular Ca^2+^ levels back towards basal values as typically observed for most Ca^2+^ signaling events since many TRP channels, including TRPV4 as discussed below, often desensitize following activation [2], [34], [35]. Since TRPV4 was observed to partially desensitize in patch clamp measurements, the sustained elevation of cytosolic Ca^2+^ is not likely the result of Ca^2+^ influx only. The apparent sustained elevation in cytosolic Ca^2+^ levels is not an artifact relating to fura 2 being quenched or sequestered because ionomycin was still able to elicit a rise in intracellular Ca^2+^ levels when added at the end of the experiments. Hence, other factors controlling cytosolic Ca^2+^ homeostasis must be invoked in the HeLa-TRPV4 overexpression system, possibly induced calcium release from stores or impaired calcium extrusion from the cytoplasm. The reason for this late effect remains to be determined in future studies.

Whole-cell patch clamp analysis provides more direct assessment of TRPV4 activity in HeLa-TRPV4 cells. As shown in Figure 4A–C, GSK101 addition leads to an early rapid activation of TRPV4 channels. The current can peak within seconds, but then rapidly decays to a pseudosteady state over approximately 3 minutes. The early rapid activation of the channel likely reflects a Ca^2+^-induced sensitization of the channel similar to that shown by others [36], [37]. The subsequent decay of the TRPV4 currents over the following 3 minutes reflects an early, but partial, desensitization of the channel in the continued presence of GSK101. This desensitization can be due to numerous factors, including channel endocytosis from the plasma membrane (see below), but could also be related to other factors such as a Ca^2+^-induced or Ca^2+^-calmodulin induced desensitization as noted for other channels, including TRP channels [35]–[38]. However, the whole cell recording of HeLa-TRPV4 cells in Ca^2+^-free extracellular solution showed an even larger transmembrane current with the similar activation pattern and outward rectification under GSK101 stimulation (Figure 4D–E). This is likely due to Na^+^ influx as TRPV4 is a non-selective cation channel permeable to Ca^2+^ and Na^+^. Since the GSK101 stimulated channel currents still displayed a similar desensitization to that observed in Ca^2+^ containing media, it follows that the desensitization of the channel following GSK101 stimulation is not Ca^2+^-dependent.

The desensitization of TRPV4 following GSK101 stimulation is also supported by the calcium imaging data showing that the application of ruthenium red (3 µM) and Ca^2+^-free medium following GSK101 stimulation leads to a diminished increase in intracellular Ca^2+^ concentration, but significantly more effective when applied in the early stage of GSK101 stimulation (1–3 min post activation); Ca^2+^ levels fell only minimally in the late stage (10 min activation). These studies again point to an early partial desensitization of TRPV4. Therefore, in the late stage of calcium imaging in HeLa-TRPV4 cells, the sustained intracellular Ca^2+^ concentrations above baseline is unlikely to be the direct effect of continued Ca^2+^ influx through TRPV4 channels alone, but rather the result of Ca^2+^ release from stores or diminished extrusion of Ca^2+^ from the cytosol via the plasma membrane or sarcoplasmic reticulum Ca^2+^ pumps, as heretofore described.

We directly evaluated the role of TRPV4 channel endocytosis from the plasma membrane as a determinant of TRPV4 activity. Ion channel trafficking to and from the plasma membrane is difficult to study, partly due to the difficulty in extracting pure plasma membrane proteins and the complex steps during vesicle trafficking. In this study, we utilized a sucrose gradient-based extraction method that has produced pure plasma membrane fractions in HeLa-TRPV4 cells. Anti-alpha tubulin antibody in Western blotting is a useful marker to check whether the plasma membrane fraction is contaminated with cytosolic proteins because cytoskeletal proteins are the most common contaminants during plasma membrane extraction. Acomparison of the traditional biotin-streptavidin system with the sucrose gradient-based extraction kit shows that the sucrose gradient-based extraction method yields a highly enriched, pure, plasma membrane fraction, while the biotin-streptavidin method produces a crude plasma membrane fraction with considerable contamination of cytoskeletal proteins. However, it is worth noting that sucrose gradient-based extraction has a lower yield in the final protein quantity than biotin-streptavidin system.

Few studies have evaluated TRP channel trafficking and most have focused on translocation and insertion of TRP channel onto the plasma membrane during a stimulus. The hypothesis is that a stimulus causes channel exocytosis and insertion into the plasma membrane, resulting in an increased number of channels, leading to enhanced Ca^2+^ entry.The examples include insulin-induced TRPV2 channel insertion onto the plasma membrane in pancreatic β-cells [20], Rab11a-induced insertion of TRPV5 and TRPV6 in epithelial cells [22], [23], [39], and epidermal growth factor-induced translocation of TRPC4 and TRPC5 in HEK-293 cells overexpressing TRPC4 or TRPC5, respectively [19], [21]. However, Shukla and coworkers [24] recently reported that angiotensin induces internalization of TRPV4 in rat vascular smooth muscle cells based on confocal microscopy analysis. They also showed that in HEK-293 cells overexpressing TRPV4, angiotensin treatment induced an immediate upregulation of TRPV4 at 5 min post stimulation, followed by an approximately 50% decrease in TRPV4 plasma membrane expression over the next 1 hr using a surface biotinylation assay [24]. In the current study, we discovered that TRPV4, while being activated by GSK101, does not lead to an early insertion or elevated membrane abundance of TRPV4, but rather the activation is associated with an early and rapid downregulation of TRPV4 from the plasma membrane, i.e., TRPV4 endocytosis, within 3 min of GSK101 stimulation. A pure plasma membrane protein extraction method, as well as normalized TRPV4 expression using pan-cadherin as loading control for densitometry, was utilized to ensure the accuracy of the observation. It is likely that the increase in intracellular Ca^2+^ serves as a negative modulator of TRPV4 membrane expression and initiates the endocytosis of TRPV4 from the plasma membrane, likely leading to reduced Ca^2+^ entry. Although the endocytosis mechanism is not known, the usual machinery for vesicle trafficking, such as Rab small GTPases, SNAREs and phosphoinisitides are likely to be involved and require further investigation [40], [41]. It is also noteworthy that Shukla et al. recently discovered a novel role of β-arrestin 1 in TRPV4 downregulation by mediating ubiquitination[24]. This expands the endocytosis pathways of ion channels, and points to potential mechanisms of TRPV4 downregulationin the plasma membrane under GSK101 stimulation for future study.

To provide a more in depth assessment of the physical determinants of TRPV4 activation, we attempted to decipher the mechanism of GSK101 activation/desensitization of TRPV4 channels using a FRET analysis to evaluate the physical association among subunits of the TRPV4 homotetramer. Alterations in the physical associations between channel subunits are thought to play an important role in activation/modulation of TRP channels [27], [42]–[44]. Indeed, channel subunit dissociation may be an important regulatory process underlying channel inactivation for some TRP channels as shown for TRPP2 [29], [30]. A recent study by Shigematsu et al confirms that TRPV4 forms functional homotetramers[33] while others have shown that TRPV4 heterotetramers can form [26], [27] and that subunit mixing among various TRP channel family members can lead to altered channel functions [26]–[28]. Our FRET analysis utilizes TRPV4-mCerulean and TRPV4-mVenus as donor and acceptor to evaluate homotetramericassembly. The results show that GSK101 does not lead to an increased FRET transfer efficiency between the TRPV4 subunits, or more specifically at the tagged C-termini, on the plasma membrane, indicating that the GSK101-induced TRPV4 activation does not involve theassembly of TRPV4 subunits into new homotetrameric structures. Likewise, with continued exposure to GSK101, the desensitization of TRPV4 also does not lead to a change in plasma membrane FRET efficiency, indicating that desensitization does not appear to involve a dissociation of TRPV4 subunits from homotetrameric structures. We conclude that the activation and desensitization of TRPV4 following GSK101 stimulation does not appear to involve alterations in the homotetrameric structure of the TRPV4 channel at the plasma membrane. Activation of the channel at the plasma membrane would, therefore, appear to reflect activation of existing channel structures with conformational changes within the homotetrameric structure that lead to channel opening. With the 3D structure of TRPV4 available [33], it will be interesting to define the changes within the homotetrameric structure itself that lead to alterations in channel gating, potentiation, and desensitization.

## Materials and Methods

### Cloning and chemicals

Mouse TRPV4 coding sequence (Accession #NM_022017) was inserted between *Sal I* and *Bam HI* sites of mCerulean-N1 and mVenus-N1 vectors (Roger Tsien laboratory, University of California at San Diego), so that the mCerulean or mVenus fusion protein was at the C-terminus of recombinant TRPV4 protein. To simplify the terminology in this work, TRPV4-mCerulean stands for mCerulean-N1-TRPV4 and TRPV4-mVenus stands for mVenus-N1-TRPV4. Both plasmids went through sequencing to verify the correct DNA sequences. The recombinant TRPV4-mCerulean and TRPV4-mVenus proteins were also characterized by western blots using both anti-TRPV4 antibody (Alomone Labs, Jerusalem, Israel) and anti-GFP antibody (AbCam, Cambridge, MA) (data not shown). The mCerulean or mVenus fusion protein added to the recombinant TRPV4 did not alter the amino acid sequence at the C-ternimus of TRPV4, therefore leaving the epitopes intact for antibody recognition.

GSK1016790A (GSK101) was either a gift from GlaxoSmithKline or purchased from Sigma-Aldrich (St. Louis, MO). GSK101 was dissolved in DMSO at 10 mM stock concentration and stored in aliquots at −20°C. It was then diluted into various working concentrations right before use.

### Cell culture and transfection

HeLa cells (human cervical cancer) were grown in RPMI medium supplemented with 10% FBS, 1 IU/ml penicillin and 1 µg/ml streptomycin in a 37°C and 5% CO2 incubator. HeLa cells were seeded onto glass coverslips or tissue culture dishes within 24 hrs of transfection, then an Effectene kit (Qiagene, Valencia, CA) was used to transfect HeLa cells with different plasmids. Cells were used for various experiments 20 to 24 hrs post transfection.

### Calcium imaging

HeLa-TRPV4 cells grown on glass coverslips were loaded with 2 µM fura-2/AM for 1 hr at RT, then washed in the isotonic MBSS buffer (containing 140 mMNaCl, 5.4 mMKCl, 0.5 mM MgCl_2_, 0.4 mM MgSO_4_, 3.3 mM NaHCO_3_, 2 mM CaCl_2_, 5.5 mM glucose, 10 mM HEPES, pH 7.4). The coverslip was attached to a perfusion chamber, which was then attached to the stage of an InCyt imaging workstation (Intracellular Imaging) for imaging of intracellular Ca^2+^ levels [5], [13]. For HeLa-TRPV4 cells, only those cells positively identified for TRPV4-mVenus expression with epifluorescence at 488 nm wavelength were selected for imaging. Cells were bathed in isotonic MBSS buffer and 10 to 20 cells of interest were selected using a freehand ROI (Regions of Interest) drawing tool. Then the cells were treated with 10 nM GSK101 and recorded for different periods. Ca^2+^-free experiments were carried out by washing away the Ca^2+^-containing MBSS with the Ca^2+^-free MBSS (MBSS without CaCl_2_, with the addition of 1 mM EGTA). Intracellular Ca^2+^ was calculated using the fura-2 fluorescence ratio method (excitation at 340 over 380 nm, emission at 510 nm) which was converted to intracellular Ca^2+^ concentration as described by Gadzikowska et al. [45] using the calibration methods as done before [5], [13], [46].

To better compare changes in [Ca^2+^]_i_ between methods of TRPV4 inhibition, in some studies the intracellular Ca^2+^ concentration was normalized for each data point. Delta _GSK101_  =  [Ca^2+^]_i_ with GSK101 – [Ca^2+^]_i_ at basal level. Delta _inhibitor_  =  [Ca^2+^]_i_ with inhibitor – [Ca^2+^]_i_ at basal level. Percentage inhibition  =  [(Delta _GSK101_ - Delta _inhibitor_)/Delta _GSK101_] x 100%.

### Electrophysiology

Patch electrodes with a resistance of ≈2 MΩ were pulled from borosilicate micropipettes (Sutter Instrument Company, Novato, CA) using a micropipette puller (P-97 Sutter Instrument Co., Novato, CA) [5]. HeLa cells transfected with TRPV4-mVenus were identified using a combination of epifluorescence illumination and differential interference contrast optics (20X–40X) on an inverted Axiovert 200M microscope (Carl Zeiss, Germany). Cells were recorded in the whole-cell configuration using an EPC-10 amplifier (HEKA Instruments, Germany). The junction potential was 13 mV (n = 14), which was compensated using Pulse software. After whole-cell configuration was established, the cell membrane capacitance (32.7±2.4 pF) and series resistance (7.1±0.8 MΩ, n = 14) were electronically compensated. Whole-cell currents were not normalized to capacitance. To determine the exponential decay of the currents, data were fitted with SigmaPlot using an exponential decay function Y =  y0 + Ae^-t/τ^, where Y is the current at time t, y0 is the residual current.

All experiments were performed at room temperature (≈25°C). Signals were filtered at 1 kHz, digitized at 10 kHz, and acquired using the Pulse program (HEKA Instruments, Germany). The Ca^2+^-containing extracellular solution consisted of 136 mMNaCl, 5.4 mMKCl, 0.5 mM MgCl_2_, 0.4 mM MgSO_4_, 3 mM NaHCO_3_, 2 mM CaCl_2_, 5 mM glucose and 10 mM HEPES (pH 7.4, 300 mOsm). The Ca^2+^-free extracellular solution consisted of 145 mMNaCl, 1 mM MgCl_2_, 10 mM HEPES, 10 mM glucose and 1 mM EGTA (pH 7.4, 300 mOsm). The pipette internal solution contained 20 mMCsCl, 100 mM Cs aspartate, 1 mM MgCl_2_, 5 mM EGTA, 10 mM HEPES, 1 mM Mg-ATP, and 0.1 mM Na-GTP (pH 7.2, 290 mOsm). The holding potential was −90 mV for most experiments. The current-voltage relation was determined using a voltage ramp protocol from −100 to 100 mV over 200 msec.

GSK101 stock in DMSO (10 mM) was diluted 1∶1000 in extracellular solution just before use and held in a series of independent syringes connected to an array of corresponding fused silica columns (inner diameter, 200 µm). The exchange of solutions was achieved by rapidly by shifting the tubes horizontally with a micromanipulator. The distance from the column mouth to the cell examined was about 100 µm. Cells in the recording chamber were continuously bathed in the extracellular solution. Each solution was delivered to the recording chamber by gravity.

### Western blotting and densitometry

HeLa-TRPV4 cells were grown on 10-cm tissue culture plates until ready for harvest. The cells were rinsed twice with cold PBS, and scraped off of the plates using a spatula. Then a sucrose gradient-based membrane protein extraction kit (Biovision, Inc., Mountain View, CA) was utilized to purify plasma membrane proteins. Whole cell lysates were the intermediate products before the plasma membrane fraction was purified from the sucrose gradient. The final pellets containing pure plasma membrane proteins were achieved at the end of the extraction process. The resultant pellets were solubilized in a modified RIPA buffer (0.1% Na deoxycholate, 0.01% SDS, 1% NP-40 and 20 mM Mg acetate in PBS) containing protease inhibitor cocktail (Sigma-Aldrich, St. Louis, MO). Protein concentrations were measured using the BCA method. Standard Western blotting (AbCam, Cambridge, MA) was performed to characterize and quantify TRPV4 channel expression. Primary antibodies used in this work include anti-TRPV4 (Alomone Labs, Jerusalem, Israel) for detecting TRPV4 as well as TRPV4-mVenus/Cerulean, anti-pan cadherin antibody (AbCam, Cambridge, MA) as internal loading control, and anti alpha-tubulin antibody (Sigma-Aldrich, St. Louis, MO) for verifying the purity of plasma membrane preparation. Densitometry (Image J 1.42q, NIH) was utilized to quantify the abundance of a TRPV4 channel in the plasma membrane under different treatments. Normalized TRPV4 channel quantity was achieved by calculating the ratio of TRPV4/pan cadherin, as pan cadherin was the loading control for all the plasma membrane samples. All experiments were repeated 4 to 5 times.

### Fluorescent Resonance Energy Transfer (FRET)

FRET microscopy was performed as described by Kenworthy group [5], [47], [48]. HeLa cells grown on glass coverslips were co-transfected with TRPV4-mCerulean and TRPV4-mVenus with fluorescent fusion protein at the carboxyl terminus of the TRPV4 insert. Twenty hours after the transfection, the cells were washed in PBS and fixed in 4% paraformaldehyde for 20 minutes at room temperature. The coverslips were then washed and mounted onto glass slides using Prolong mounting media (Invitrogen). Images were captured on a Nikon A1 confocal microscope. Three pre-bleach images of both mCerulean and mVenus were acquired (mCerulean_pre_ and mVenus_pre_, respectively). Acceptor photobleach was achieved by laser excitation at 514-nm set at 100% transmission for duration of 30 seconds (1 pulse/second). Then three post-bleach images of both mCerulean and mVenus were acquired (mCerulean_post_ and mVenus_post_, respectively). Regions of interest (ROI) were manually selected over the perinuclear region, designated as perinuclear (PN), or plasma membrane region, designated as membranous (M). A background ROI was also selected. The fluorescent intensity of the ROI was measured, background-subtracted and exported from the NIS software as an Excel file. The average of the pre-bleach and post-bleach fluorescence intensities were calculated from three images. The FRET efficiency was calculated as E =  (mCerulean_post_ - mCerulean_pre_)/mCerulean_post_. A chart graph was generated after averaging 50 to 80 ROI measurements from three sets of GSK101 time-course experiments.

### Statistical Analysis

All data are presented as mean ± SE. Student t-test was used to compare the means between two treatment groups. P-values less than 0.05 were considered statistically significant.

A GSK101 dose-response curve was generated using the mean response (n = 4–6) at various GSK101 concentrations in SigmaPlot 10.0. The sigmoidal dose-response curve employing EC_50_ and Hillslope was chosen for calculation.
